# Valorization of frying oil waste for biodetergent production using *Serratia marcescens* N2 and gamma irradiation assisted biorecovery

**DOI:** 10.1186/s12934-022-01877-3

**Published:** 2022-07-30

**Authors:** Nora M. Elkenawy, Ola M. Gomaa

**Affiliations:** 1 Drug Radiation Research Department, National Center for Radiation Research and Technology (NCRRT), Egyptian Atomic Energy Authority (EAEA), Cairo, Egypt; 2 Radiation Microbiology Department, National Center for Radiation Research and Technology (NCRRT), Egyptian Atomic Energy Authority (EAEA), Cairo, Egypt

**Keywords:** Biosurfactant, *Serratia marcescens*, Oil valorization, Biorecovery, Biodetergent, Gamma irradiation

## Abstract

**Background:**

The complexity, toxicity and abundance of frying oil waste (FOW) render it difficult to be degraded biologically. The aim of the present work was to valorize FOW and investigate the potential use of the produced biosurfactant by *Serratia marcescens* N2 (Whole Genome sequencing accession ID SPSG00000000) as a biodetergent.

**Results:**

*Serratia marcescens* N2 demonstrated efficient valorization of FOW, using 1% peptone, 20% FOW and 8% inoculum size. Gene annotation showed the presence of serrawettin synthetase indicating that the produced biosurfactant was serrawettin. Zeta potential and Fourier Transform Infrared (FTIR) spectroscopy indicate that the biosurfactant produced was a negatively charged lipopeptide. The biosurfactant reduced the surface tension of water from 72 to 25.7 mN/m; its emulsification index was 90%. The valorization started after 1 h of incubation and reached a maximum of 83.3%. Gamma radiation was used to increase the biosurfactant yield from 9.4 to 19.2 g/L for non-irradiated and 1000 Gy irradiated cultures, respectively. It was noted that the biorecovery took place immediately as opposed to overnight storage required in conventional biosurfactant recovery. Both chemical and functional characteristics of the radiation induced biosurfactant did not change at low doses. The produced biosurfactant was used to wash oil stain; the highest detergency reached was 87% at 60 °C under stirring conditions for 500 Gy gamma assisted biorecovery. Skin irritation tests performed on experimental mice showed no inflammation.

**Conclusion:**

This study was able to obtain a skin friendly effective biodetergent from low worth FOW using *Serratia marcescens* N2 with 83% efficient valorization using only peptone in the growth media unlike previous studies using complex media. Gamma radiation was for the first time experimented to assist biosurfactant recovery and doubling the yield without affecting the efficiency.

**Supplementary Information:**

The online version contains supplementary material available at 10.1186/s12934-022-01877-3.

## Background

Biocircular and green (BCG) economy has been integrated with the realization of the 17 sustainable development goals (SDGs). Sustainability oriented society would entail the integration of multidisciplinary stakeholders who all work to pave the way for such a transformation to meet social, economic and ecological goals [1]. The basic concept of BCG economy is to utilize waste as a new resource to obtain value-added products, thus addressing sustainability [2]. One of the problematic wastes that have been perceived lately as a new resource is the oil waste generated from frying foods. FOW results from the fast food industry and is considered one of the main worldwide solid organic urban waste.FOW production is expected to reach 2.5 × 10^9^ tons worldwide by 2025 [3]. Europe produces a range from 100,000 to 700,000 tons per year [].

Conventional methods of disposal as direct disposal in sewage systems [4] can lead to other problems such as clogging the drains due to the viscosity of pre-used oil. FOW is mixed with food waste for decomposition in landfills, they release greenhouse gases [], and this is expected to play a role in climate change. FOW is commonly recycled into other value-added products such as soap, a very simple transformation that is attracting the attention of households and start-up companies in Egypt. Using microorganisms to utilize and transform FOW and convert it to value-added product such as biosurfactant is another attractive solution [5–7].

Biosurfactants are prominent amphiphilic compounds that consist of hydrophilic and hydrophobic moieties. The hydrophobic part consists of a fatty acid or fatty alcohol chain with atoms ranging from C8 to C18. The hydrophilic component can be a carbohydrate or a protein. The diverse structural variations imply a variety of physicochemical properties [8]. Biosurfactants are perceived as the next generation multifunctional biomolecules that can be used for an array of applications such as medicine, industry, cosmetics and bioremediation. Yet, biosurfactant production faces limitations that entail low microbial productivity and high cost for down streaming process and raw material availability [9, 10]. Applying strategies for bioprocess upscale can be considered a counteract approach to those limitations. The use of waste as a low cost carbon source and computational tools for media selection and genetic engineering are among the methods for optimizing biosurfactant production [11–13]. In addition to that, finding solutions to the down streaming process is imperative to the commercial production of biosurfactants. The reverse aqueous extraction method and calcium precipitation were used as novel approaches to polish biosurfactant production [12].

But apart from using the optimal media components for production, the amount released is dependent on the biosurfactant mobility from within the cell to the cultivation media via the cell membrane. Suryawanshi et al. [14] studied different biosurfactant extraction approaches such as organic solvent, precipitation using different chemicals and pasteurization and found the most efficient extraction method was the organic solvent extraction. This method of extraction requires overnight storage at 4 °C. From this standpoint; gamma radiation can be considered an effective tool to release the biosurfactant from bacterial cells. Gamma radiation was previously used to permeabilize the cell wall of gram positive bacteria to help release biomolecules to the media [15].

Therefore, the present study aims to valorize FOW to produce biosurfactants, apply factorial design to reach optimal productivity and use gamma radiation to assist in the biosurfactant down streaming process. Structural and functional changes post gamma radiation use will be assessed in terms of its application as a biodetergent.

## Results

### Screening of different *Serratia marcescens* strains for biosurfactant production

Five *Serratia marcescens* strains were tested for their biosurfactant production. The results in Table 1 show that although the five pigmented strains produced biosurfactants, yet their surface tension varied ranging from 29.8 to 38.9 mN/m. *S. marcescens* N2 biosurfactant reduced the surface tension of water from 72 to 29.8 mN/m and therefore, it was chosen as the candidate strain.

**Table 1:** Screening of different *Serratia marcescens* strains for biosurfactant production, comparison was performed using surface tension

Strain	Surface tension (mN/m)
*Serratia marcescens* N2	29.8
*Serratia marcescens* MN2	36.6
*Serratia marcescens* MN3	33.3
*Serratia marcescens* MN4	38.9
*Serratia marcescens* MN5	33

### Optimization of production using *Serratia marcescens* N2 using factorial design

**Fig. 1:**
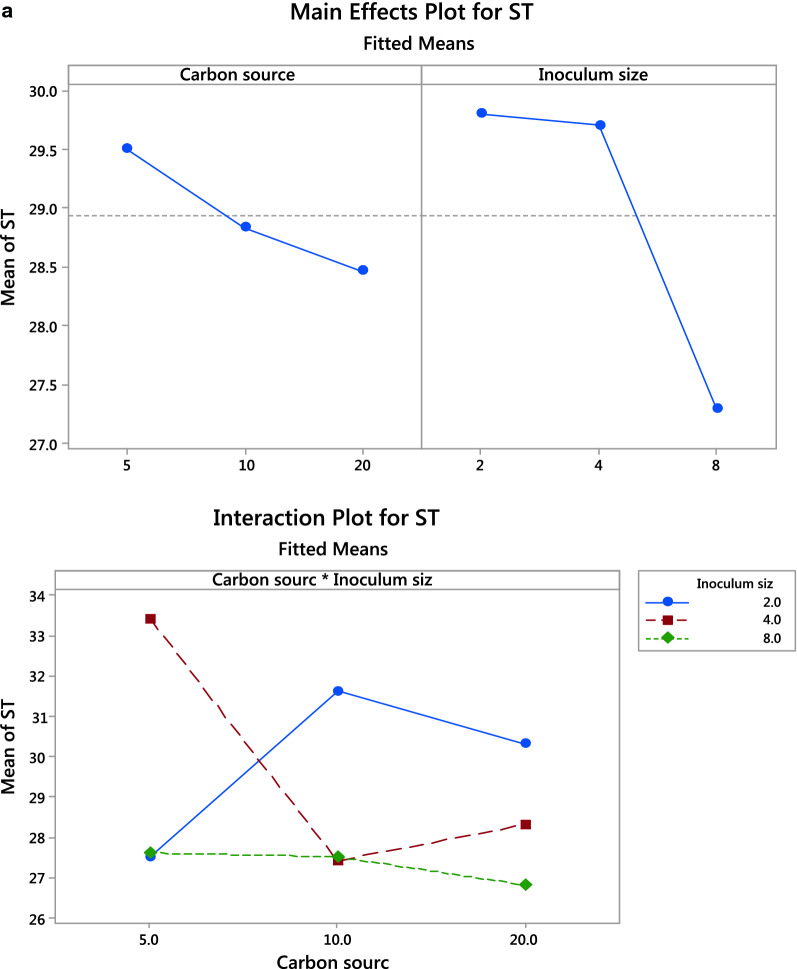

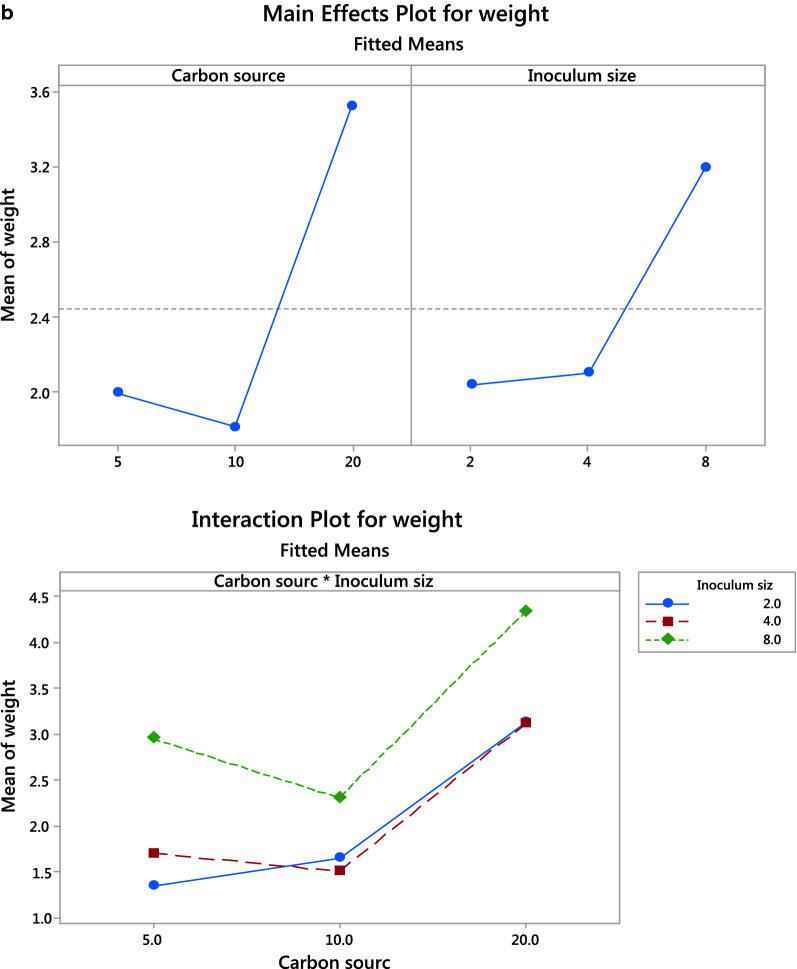
**a**: Main effects and interaction plots for surface tension of produced biosurfactant under conditions applied through factorial design. **b** Main effects and interaction plots for surface tension and weight of produced biosurfactant under conditions applied through factorial design

A 2 factors 3 levels full factorial design was used to test *S. marcescens* N2 valorization of FOW into produced biosurfactant and the possibility of increasing the amount of used oil to maximize valorization. Results obtained in Fig. 1a&1b show that 20% carbon source and 8% inoculum size resulted in the lowest surface tension (26.8 mN/m) and highest biosurfactant wet weight (4.34 g).

### Characterization of *Serratia marcescens* N2 biosurfactant and oil valorization

Genome annotation for this bacterium shows the presence of serrawettin synthetase gene that is responsible for serrawettin production. Table 2 represents the identity (%) of serrawettin synthetase produced by *S. marcescens* N2 as compared to other genes present in public database.

**Table 2:** Identity percentage of serrawettin synthetase as compared to other genes in the public database

Gene from NCBI library	% identity
*Serratia marcescens* swrW gene for putative serrawettin W1 synthetase, complete cds GenBank: AB193098.2 GenBank Graphics > AB193098.2 Serratia marcescens swrW gene for putative serrawettin W1 synthetase, complete cds	99%
*Serratia marcescens* strain N4-5 serrawettin W1 synthetase gene, partial cds GenBank: EF122074.1 GenBank Graphics > EF122074.1 Serratia marcescens strain N4-5 serrawettin W1 synthetase gene, partial cds	100%
*Serratia marcescens* strain ATCC 274 serrawettin W1 synthetase gene, partial cds GenBank: EF122077.1 GenBank Graphics > EF122077.1 Serratia marcescens strain ATCC 274 serrawettin W1 synthetase gene, partial cds	100%
*Serratia marcescens* strain A88copa13 serrawettin W2 gene, partial cds GenBank: JX667980.1 GenBank Graphics > JX667980.1 Serratia marcescens strain A88copa13 serrawettin W2 gene, partial cds	No match
*Serratia liquefaciens* serrawettin synthase (swrA) gene, partial cds GenBank: AF039572.1 GenBank Graphics > AF039572.*1 Serratia liquefaciens* serrawettin synthase (swrA) gene, partial cds	No match

**Fig. 2:**
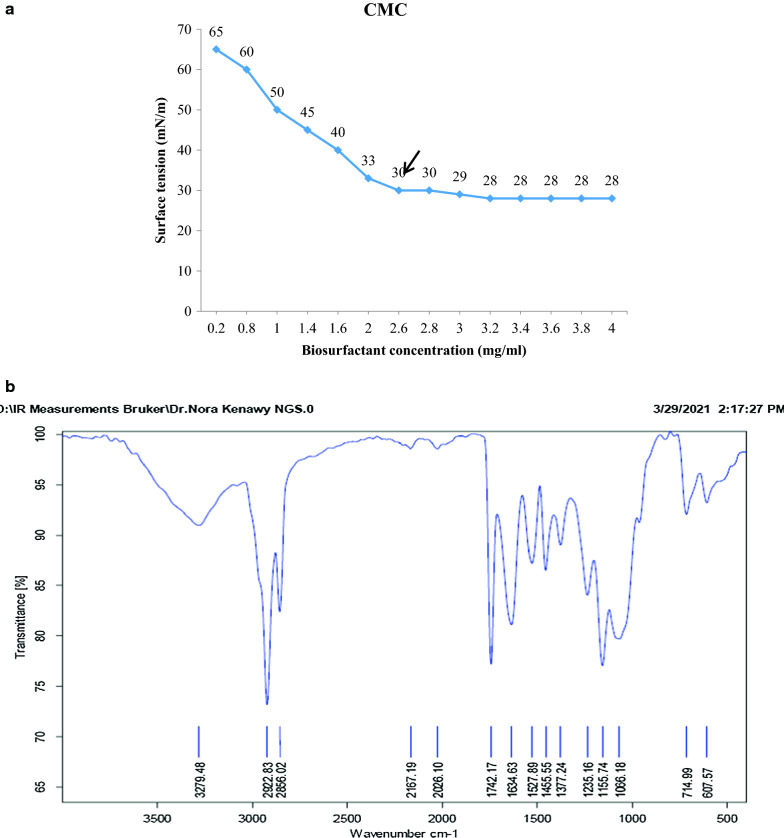
**a**: CMC of the crude isolated biosurfactant. **b** ATR-FTIR spectrum of *Serratia marcescens* N2 biosurfactant

**Fig. 3:**
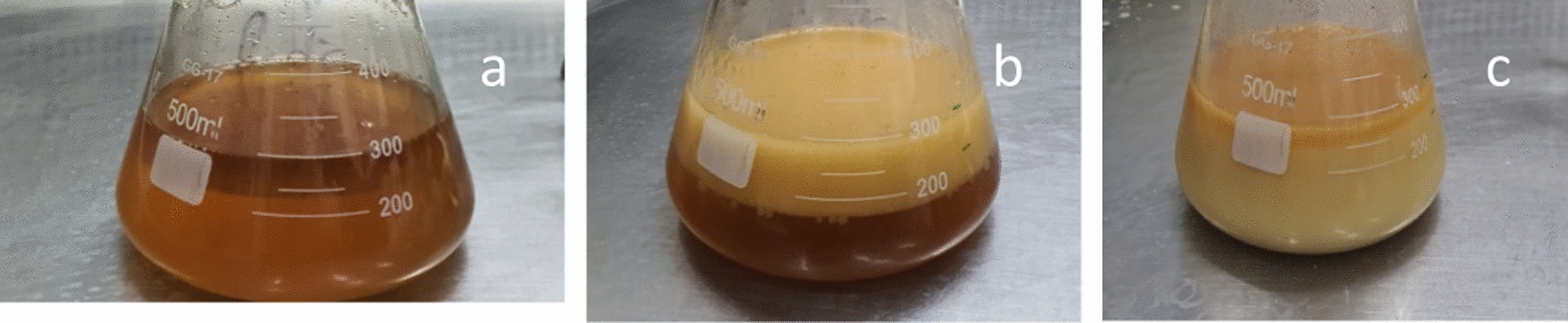
Oil valorization steps for *Serratia marcescens* N2 at zero time **a**, 1 h **b** and 6 days **c** of incubation

In addition, the zone of oil displacement activity was recorded as positive (Table 3 and Additional file 1: Fig. S1). The partially purified biosurfactant has critical micelle concentration (CMC) of 2.6 mg/ml (Fig. 2a) and exhibited negative charge of value -22 mV (Table 3). The biosurfactant was analyzed through ATR-FTIR (Fig. 2b) for determining the functional groups in their backbone. The biosurfactants showed broad and sharp peaks in the region of 3310–1657 cm^−1^ represent NH stretching and C-O-N indicating the presence of peptides, 2922 and 2666 cm^−1^ represent CH_2_ and CH_3_ groups. A strong peak at 1742 cm^−1^ suggested the presence of a carbonyl group attached to electron-withdrawing groups. The bands at 714 and 607 cm^−1^ correspond to aromatic monosubstituted ring., EI_24_ was 90% and surface tension dropped from 72 mN/m (standard ST for water) to 25.7 mN/m (Table 3). The oil valorization was calculated to be about 83.3%. with only 10 mls remaining from the primary used volume 60 ml. Additional file 1: Fig. S2 Fig. 3 clearly shows the emulsification of oil after 1 h (3b) indicating the start of production of biosurfactant and after 6 days (3c) almost complete emulsification compared to zero time (3a).

**Table 3:** Characterization of biosurfactant

Feature	Surface tension (mN/m)	Oil spreading	EI_24_%	Zeta potential	CMC (mg/ml)
Value	25.7	+ + +	90	−22	2.6

### Effect of gamma radiation as a tool for biosurfactant release from *Serratia marcescens* N2 cells on some biosurfactant structure and characteristics

**Fig. 4:**
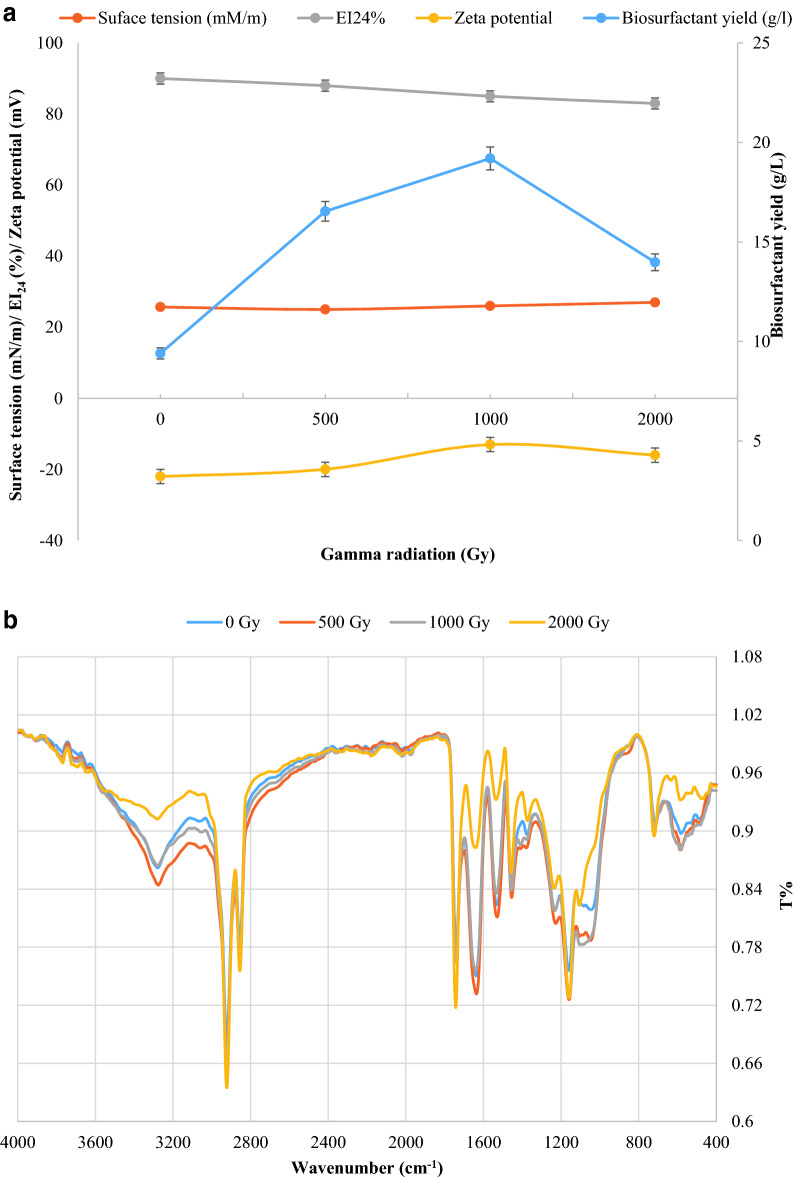
**a** Biosurfactant yield, surface tension, EI_24_% and Zeta potential of biosurfactant recovery after exposing *Serratia marcescens* N2 cultures to gamma irradiation. **b** ATR-FTIR spectrum of *Serratia marcescens* N2 biosurfactant structure after extraction using different gamma radiation doses

To avoid the multiple steps, low dose gamma radiation was used as a single step, the cell cultures were exposed to different doses of gamma radiation and the yield was calculated as 9.4, 16.5, 19.2 and 13.9 g/l for 0, 500, 1000 and 2000 Gy exposed cell cultures. The results in Fig. 4a show that emulsification index, surface tension and zeta potential were almost the same and no evident functional changes while FTIR spectrum showed changes in broadness, sharpness and intensity of peaks at 3282, 1635, 1049 and 578 cm^−1^ for 2000 Gy irradiation assisted biosurfactant recovery (Fig. 4b).

### Biodetergency and skin irritation test

**Fig. 5:**
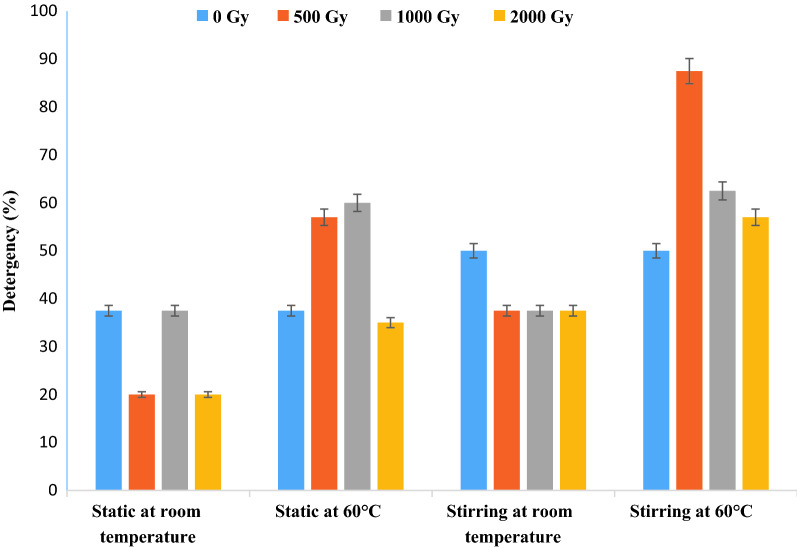
Effect of using gamma irradiation on *Serratia marcescens* N2 biosurfactant detergency (%) under static and stirring conditions at room temperature and 60 °C

**Fig. 6:**
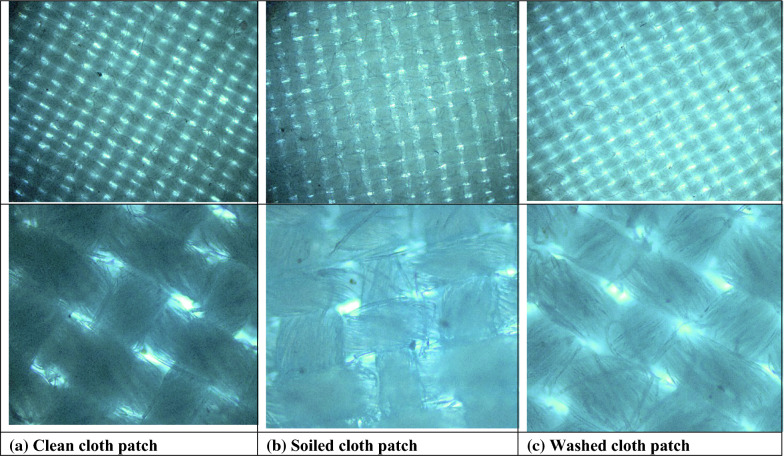
Optical images at magnification 25X (upper row) and 100X (lower row) representing clean cloth patch **a**, soiled cloth patch **b** and soiled patch washed with *Serratia marcescens* N2 500 Gy assisted biosurfactant at 60 °C and stirring conditions **c**)

**Fig. 7:**
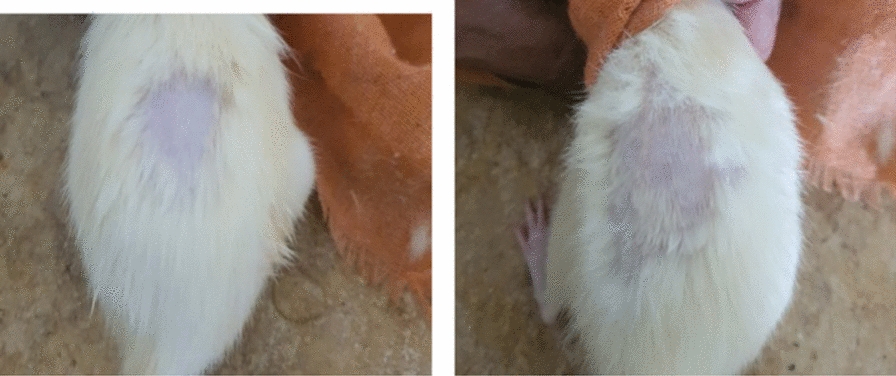
Images representing the effect of *Serratia marcescens* N2 biosurfactant on skin of mice after 24 h application of cotton pad soaked with 1 ml of biosurfactant (**b**) as compared to water soaked cotton pad (**a**)

The results shown in Fig. 5 represent crude biosurfactant that was used to clean fabric soiled with oil. The results show that the highest detergency of 87.5% was obtained after 500 Gy radiation assisted biorecovery this was followed by 62.5% for 1000 Gy and 57% for 2000 Gy radiation assisted biorecovery as compared to 50% for non-irradiated biosurfactant when the fabric was washed at 60 °C under stirring conditions. Images in Fig. 6 show that washing the soiled cloth patch with 500 Gy assisted biosurfactant at 60 °C under stirring conditions did not affect the general threading of the fabric. Figure 7 shows that all tested mice showed no redness or spots.

## Discussion

Screening of biosurfactant production using different *S. marcescens* strains was performed as an initial step. *S. marcescens* N2 with the highest production was identified as prodigiosin hyper producing strain in a previous study [16]. Variation between *S. marcescens* isolates could be intrinsic within every strain capable of biosurfactant production. The initial screening was done using clean frying oil to produce biosurfactant in a minimal challenging condition to assess their production capability avoiding the complex composition of the FOW that might be a challenge for bacterial production.

Usually, complex or multicomponent media is used to produce biosurfactants [17, 18], but in the present study, a simple media of peptone water was the base for biosurfactant production. Peptone was reported to be a suitable organic nitrogen source for biosurfactant since the amino acids present buffer the pH and therefore maintain the lipase activity which was reported to aid in emulsification of oil throughout the production process [7]. Carbon source and inoculum size were chosen as two key factors that control *Serratia marcescens* N2 biosurfactant production. Although it was expected that the experimental design would reflect on the variation significance of the results it was not significant. However, the experimental design analysis showed a significant interaction between Inoculum size and FOW expressed as a direct relationship [19]. This result indicates that *S. marcescens* N2 can withstand and utilize a high content of FOW and transform it into a biosurfactant. This could be attributed to either oil utilization as a carbon source or as an inducer for biosurfactant production by inducing biosurfactant biosynthetic clusters [20]. Balancing added nutrients and microbial growth is needed to reach good biosurfactant production [21].

On the other hand, during this experiment, it was noted that the strain lost its pigment during the process which indicates that it interfered with prodigiosin production pathway, resulting in shut down and directing the production to biosurfactant only. The production of secondary metabolites is highly dependent on cultivation conditions or environmental factors. Serratia metabolite production is regulated via quorum sensing (QS) system that influences cell-cell communication. Secondary active metabolites regulated by QS include biosurfactant and prodigiosin production [22]. Although Zhang et al. [18] reported the simultaneous production of serrawettin and prodigiosin at 30 °C, the pigment was lost at 37 °C, furthermore, the oil added to their *Serratia* culture was 10 times less than used in the current study, this is a further proof that the physiological activity of *Serratia* is highly affected by the cultivation conditions.

The whole genome sequencing of *S. marcescens* N2 was performed in a previous study [23] and the only biosurfactant identified within the genome was serrawettin synthetase, this enzyme is responsible for the synthesis of serrawettin. Serrawettin is a biosurfactant produced by *Serratia*, it can be present as Serrawettin W1 form which is a symmetric dilactone structure. The compound is composed of two serine residues, connected with two 3-hydroxydecanoic acids [24]. Thus, according to the results of the IR spectra, *S. marcescens* N2 produces a cyclic lipopeptide, this is in accordance with literature describing biosurfactants with the same characteristics. It is known that *Serratia marcescens* N2 produces serrawettin, this is a lipopeptide of low molecular weight [18, 25] also reported the production of serrawettin W1 from *Serratia marcescens* S1 that could decrease the surface tension of water to 30 mN/m and that the average surface tension for other serrawettins W1, W2 and W3 were 32.9, 33.9 and 28.8 mN/m, respectively. It is noteworthy to mention that the serrawettin produced in the current study decreased the surface tension to 26.8 mN/m (before optimization) which is lower than the reported surface tension for other studied serrawettins. Critical micelle concentration (CMC) of the produced biosurfactant value was almost half that reported by Shawkat et al. [26] and Kumari et al. [27]. A low CMC indicates that this compound can exert its effect at low concentrations [8].

Initial and consumed oil volumes were used to evaluate the oil valorization process by *S. marcescens* N2. In the present study, oil valorization reached its peak within 1–2 weeks, while oil consumption by *P. aeruginosa* PG1 was recorded to reach almost the same value after the 15 week of incubation with only 2% (v/v) crude oil added to MSM [4]. This means that our result is very promising since the initial oil added was 20% (v/v) to the culture media, suggesting that *Serratia marcescens* N2 can valorize more FOW into another product. Although gamma radiation was previously reported by some researchers to induce hyper producing bacterial mutants [28] yet in the current study, we used gamma radiation as a tool to enhance biorecovery and decrease the number of steps required for biosurfactant biorecovery. This is considered time saving in the bioprocess of biosurfactant production since the steps involves acid precipitation, overnight refrigeration and centrifugation [29]. The yield obtained is dependent on the amount of biosurfactant released in the cultivation media.

Our results indicate that *S. marcescens* N2 produces high yield of biosurfactant that increased with the exposure of cell cultures to gamma radiation, the highest yield was obtained at 1000 Gy. This result is higher than that reported by Phulpoto et al. [29] who obtained lower yields that were almost half what we obtained even after optimization of cultivation conditions. The use of gamma radiation did not incur any structural changes except in the peptide peak after exposing the cells to 2000 Gy, this change can be attributed to the effect of gamma radiation on the peptide fragment of the biosurfactant. Blanco et al. [30]reported fragmentation as one of the effects exerted by ionizing radiation on proteins. This result confirms that exposure of cell culture to 1000 Gy can result in yield increase with no structural changes or functional changes. In a previous study, 1000 Gy was enough to induce permeability of *Bacillus* sp. producing biosurfactant [15], showing that this dose is enough to induce changes without compromising the bacterial viability. Another possible role for gamma radiation can be related to increasing biosurfactant precipitation. The authors believe that the exact role of gamma radiation in increasing biosurfactant production requires more elucidation.

The detergency process occurs through the formation of micelles by surfactants that can form globules of impurities through a decrease in interface tension and with the help of electrostatic interactions between charges. However, surface-active compounds cannot completely clean dirt from the surface without the presence of other additional compounds as support, namely builders, anti-redeposition, enzymes, and other additives. Helmy et al. [31] stated that washing results that contain only 20% active ingredient in the form of biosurfactant are visually not good enough to clean stains (mild stains can be seen visually on the fabric). It is noteworthy to say that using the biosurfactant did not affect the fabric cloth threading as indicated by the captured images of clean, soiled and washed fabric cloths. Chemical surfactants were long ago reported to induce skin irritation [32], this is why the use of a biological based surfactant was sought to avoid any dermal effects. The biosurfactant under study was tested by applying it in a single dose to the skin of experimental mice, untreated skin areas of the test animal served as the control. The degree of irritation/corrosion is read and scored at specified intervals and is further described to provide a complete evaluation of the. Our results indicate that all tested mice showed no redness or spots and this confirms that biosurfactant produced by *S. marcescens* N2 is safe to use on skin in addition to its efficiency as a biodetergent.

## Conclusion

Biosurfactant production using FOW is considered a step forward in achieving a biocircular economy, it can be used as an alternative carbon source while at the same time reducing cooking oil waste. *Serratia marcescens* N2 produced serrawettin biosurfactant that decreased the surface tension of water lower than the majority of reported biosurfactants, in addition to that, it recorded high emulsification index. *S. marcescens* N2 was able to grow and valorize 20% pre-used cooking oil. The biosurfactant was used as a biodetergent without causing any skin irritation. The use of gamma radiation is considered practical since it reduced the number of extraction steps and can be easily applied in down streaming process of biosurfactant production. Using low dose gamma radiation provided a single step for biorecovery, this can be introduced to production lines as an alternative to multiple-step biorecovery. This work is to be continued for up-scale production of biosurfactant.

## Methods

### Screening of different *Serratia marcescens* strains for biosurfactant production

Biosurfactant production screening was carried out in 250 ml Erlenmeyer flasks with 100 ml of production medium containing 1% peptone and 6% clean Mix FOW with media were adjusted to pH 7.0 and autoclaved at 121 °C for 15 min. Then, they were inoculated at 2% inoculum size and incubated for 13 days at 28 °C, under orbital agitation (150 rpm).

Inoculum preparation of 5 *Serratia marcescens* strains (MN2 (KX601268,), MN3(KX601278), MN4 (KX601721), MN5(KX601170), N2 Bioproject ID PRJNA525074, Biosample ID SAMN11041520, and WGS accession SPSG00000000 Loop from plate in 10 ml Luria Bertani broth incubated for 1 h then absorbance was adjusted to 0.5 at 600 nm then 2 ml from inoculum into flask. Surface tension of the collected cell-free metabolic cultures were obtained by centrifugation at 12,000 × g for 20 min, and membrane filtration of culture media 0.22 μm. Analyses were performed at 25 °C in a Kruss tensiometer (K20 Kruss GmbH, Germany) using the Du Noüy ring method with Milli-Q water with surface tension of 72 mN/m was used to calibrate the tensiometer [20]. Oil spreading technique was performed as follows: 50 ml distilled water to Petri dish followed by 100 μl of vegetable oil to surface of water then 10 μl of cell free biosurfactant was added slowly and detected in light after 30 s [19].

### Optimization of production using factorial design

*Serratia marcescens* N2 was **t**he strain with the lowest surface tension was used in the upcoming experiments, this strain was previously deposited at DDBJ/ENA/GenBank under Bioproject ID PRJNA525074, Biosample ID SAMN11041520, and WGS accession SPSG00000000 with the annotated genome of *S. marcescens* N2 deposited in the PATRIC database under genome number 615.1488 [23]. Biosurfactant production experimental design was done using Minitab 18 software (USA) for 2 factors, 3 levels. The design of the experiment is represented in Additional file 2: Table S1. The cultivation was carried out in 250 ml Erlenmeyer flasks with 100 ml working volume. The production medium consisted of 1% peptone and carbon sources of 5, 10 and 20% vol/vol of FOW from a local restaurant in Heliopolis area, Cairo. Cultivation media were adjusted to pH 7.0 and autoclaved at 121 °C for 15 min. Flasks were inoculated with inoculum sizes 2, 4 and 8% and incubated for 6 days at 28 °C, under orbital agitation (150 rpm). Surface tension was estimated as previously described and wet biosurfactant weight was calculated as the biosurfactant weight at the end of the production process in g/l.

### Extraction and characterization of *S. marcescens*N2 biosurfactant

The biosurfactant produced by *S. marcescens* N2 after 6 days fermentation was isolated from cell-free metabolic liquid obtained by centrifuging (12,000 ×*g* for 20 min) the culture. The metabolic liquid was subjected to precipitation using Conc. HCl to get pH 2.0 and kept at 4^∘^ C overnight. It was then centrifuged at 15,000 ×*g* for 15 min and the cell-free metabolic supernatant was collected and centrifuged at 5000 ×*g* for 15 min. The supernatant obtained was discarded and the crude biosurfactant was extracted three times with a chloroform-ethanol (2: 1 v/v) mixture with vigorous shaking, The precipitate was collected, oven dried and used for analysis [33].

#### Fourier transform infrared spectroscopy (FT-IR)

The identification of functional groups in the isolated biosurfactant was carried out using Attenuated total reflectance-Fourier transform infrared spectroscopy (ATR-FTIR). The obtained biosurfactant in its dry form was scanned within the range of 4000–400 cm^−1^ using BRUKER VERTEX 70 device at NCRRT. The spectrum of the biosurfactant under study was compared to the literature.

#### Emulsification index

One ml of cell free metabolic liquid was added to one ml FOW and paraffin oil individually. Tween 80 was used as positive control and water as negative control then vortexed for 2 min and left for 24 h to measure height of emulsion layer divided by total height [34].

$${\text{EI }}{24}=\frac{Height \ of \ emulsification \ layer \ (cm)}{Total \ height \ of \ the \ mixture \ (cm)} \times 100$$

#### Zeta potential

The electrokinetic potential zeta of biosurfactant aqueous solution was analyzed using PSSNICOMP Zeta Potential/Particle Sizer 380ZLS (PSS-NICOMP, Santa Barbara, CA, USA) with 2 mg of crude biosurfactant dissolved in 1 ml of water [20].

#### Biosurfactant identification of serrawettin gene

The WGS of *Serratia marcescens* N2 was used to identify the biosurfactant gene and the results were compared to other genes deposited in NCBI database repository. Data were represented as identity percentages.

#### Oil displacement activity (ODA) and Critical Micelle Concentration (CMC) of biosurfactant

ODA was performed by adding 10 µl of cell free broth to 100 µl of engine oil that was added to a petri dish containing 40 ml distilled water. CMC of biosurfactant was determined using crude biosurfactant. Different concentrations (0.2–4 mg/ml) were prepared and surface tension was measured for each concentration. The value of CMC was obtained from the graph [20].

### Gamma radiation for biosurfactant biorecovery and assessment of the structural and functional changes

At the end of the cultivation period, the content was divided into four equal portions in clean sterile containers for gamma irradiation to test the effect of gamma radiation on biosurfactant biorecovery and yield. The irradiation process was carried out in Cobalt-60 (Co^60^) 220 gamma cell, Canada Co. Ltd. located at the National Centre for Radiation Research and Technology (NCRRT), Atomic Energy Authority, Cairo, Egypt at doses 500, 1000 and 2000 Gy. The dose rate was 1.119 kGy/ h at the time of the experiment. At the end of the gamma irradiation process, the culture filtrate was collected and biosurfactant extracted as previously described. Surface tension, emulsification index, FTIR and Zeta potential were performed as previously described. Gravimetric analysis was performed by obtaining the dry weight for the biosurfactants after oven drying.

### Oil valorization assessment

The whole content of a 300 ml flask with 1% peptone, 20% oil content and 8% inoculum size cultivated for 13 days at 150 rpm and 28 ℃. The remaining oil content at the end of the experiment was measured to evaluate the valorization of using FOW to produced biosurfactant. The dry cell weight was measured to assess bacterial ability to valorize oil into biosurfactant. The consumed oil was divided by the initial oil to obtain the valorization percentage $\frac{\mathrm{consumed oil}}{\mathrm{initial oil}}\times 100$ [4].

### Application of biosurfactant as washing detergent

#### Washing experiment

Pieces of dry cotton cloth were cut into 2 × 2 cm pieces and each piece was stained with 0.3 ml FOW. The pieces were left for 15 days to stabilize the stain as a challenge for stain removal, and their precise weights were recorded before staining, after staining and after washing.

The stained cotton cloths were washed using biosurfactant released from cells after culture exposure to 500, 1000 and 2000 Gy. The results were compared to non-irradiated biosurfactant producing culture at 1 g/L tap water under both static and stirring in a water bath at 50 rpm and 30 ℃ and 60 ℃ for 1 h. After washing, the pieces were rinsed in water for half an hour and dried at ambient temperature to a constant weight. The removal percentage of each stain was calculated using the precise weights of the pieces before and after washing [34, 35].

$$Detergency (\%)=\frac{C-B}{A-B} \times 100$$

where *A* is the weight of soiled cloth, *B* is the weight of white cloth, and *C* is the weight of a soiled cloth after washing [36].

#### Optical microscopy

Optical images of clean, soiled and soiled clot patches washed with 500 Gy assisted biosurfactant at 60 °C under stirring conditions were captured using AX10 Zeiss light microscope coupled with Axiocam 105 color (Germany) at NCRRT. Images representing the fabric threading were captured at 25X and 100X magnification.

#### Skin irritation test

Acute dermal irritation test was performed Co. based on OECD/OCDE404 method after the approval of Research Ethics Committee at the National Research Center or Research and Technology (REC-NCRRT) with serial number 47 A/21, the result was expressed in terms of primary irritation index (PII). Irritation scores for erythema, eschar and edema formation at 1, 24, 48 and 72 h after patch removal were summed up and divided by the number of observations, to obtain the individual PII. For the calculation of PII, all individual PII's were summed up and divided by the number of animals used during the test. The detailed information on the experimental procedures of each test was described in OECD [37].

## Supplementary Information

**Additional file 1: Fig S1.** Oil displacement activity (ODA) before biosurfactant addition (a) after biosurfactant addition (b). **Fig S2.** Remaining FOW at the end of biosurfactant production experiment.

**Additional file 2: Table S1.** Design of experiment.

## Data Availability

The obtained data will be available upon request.
