# Supplementary material for: Valorization of frying oil waste for biodetergent production using Serratia marcescens N2 and gamma irradiation assisted biorecovery
Source: Microb Cell Fact. 2022 Jul 30;21:151. doi: 10.1186/s12934-022-01877-3 (PMC9338678; doi:10.1186/s12934-022-01877-3)
Supplement: Supplementary file 1 — Additional file 1: Fig S1. Oil displacement activity (ODA) before biosurfactant addition (a) after biosurfactant addition (b). Fig S2. Remaining FOW at the end of biosurfactant production experiment. [file 12934_2022_1877_MOESM1_ESM.docx]

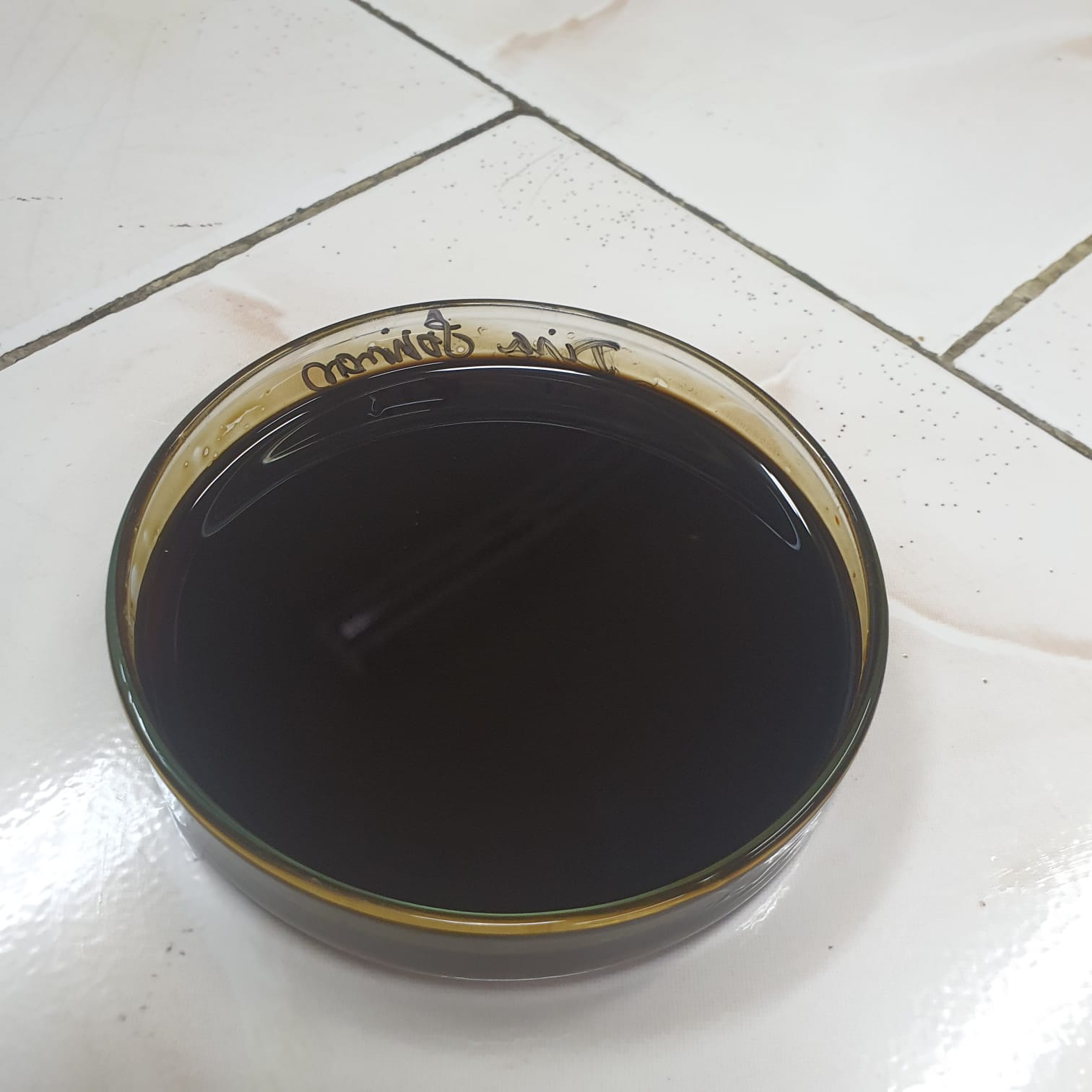

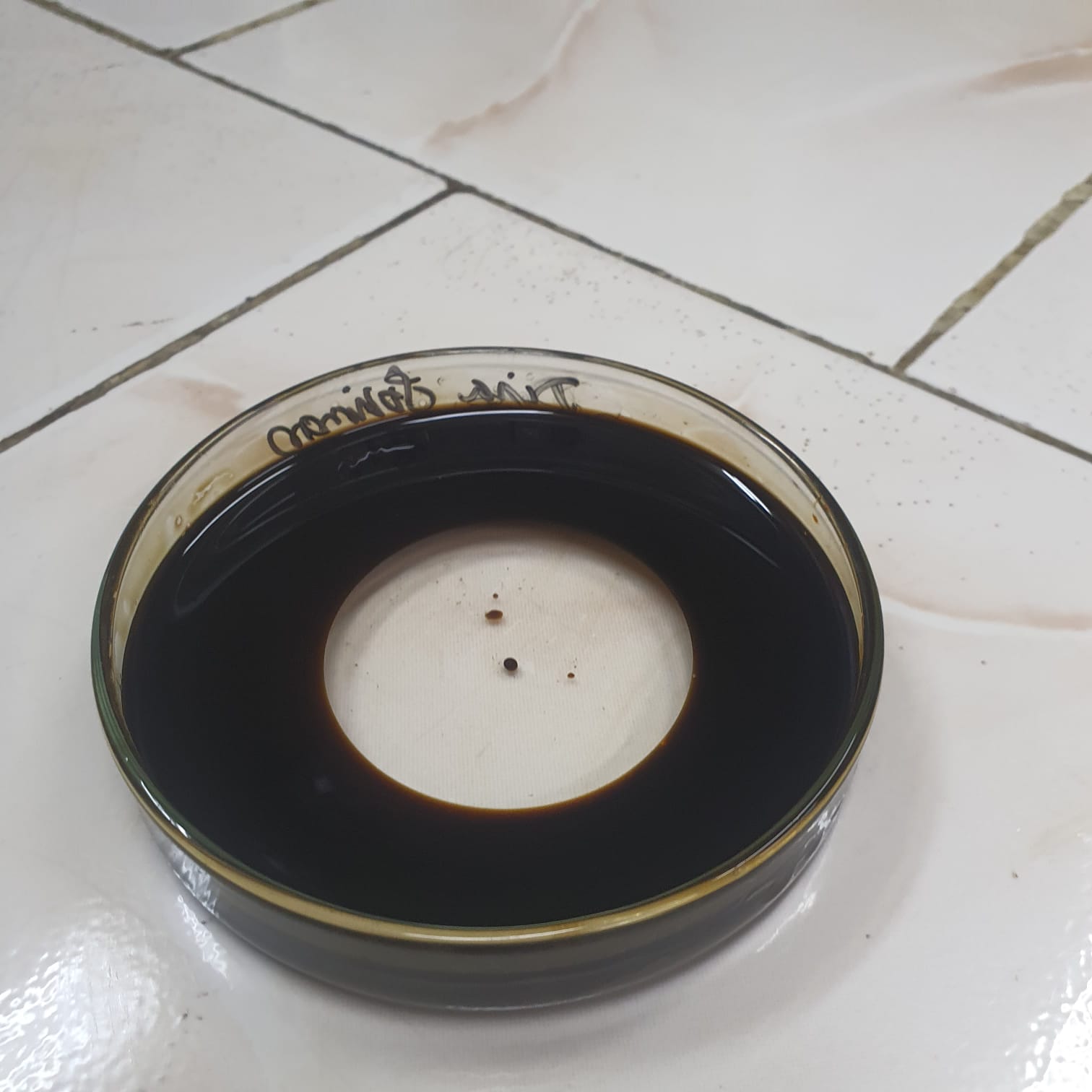


b

a

Fig S1: **Oil displacement activity (ODA)**

**before biosurfactant addition (a) after biosurfactant addition (b)**


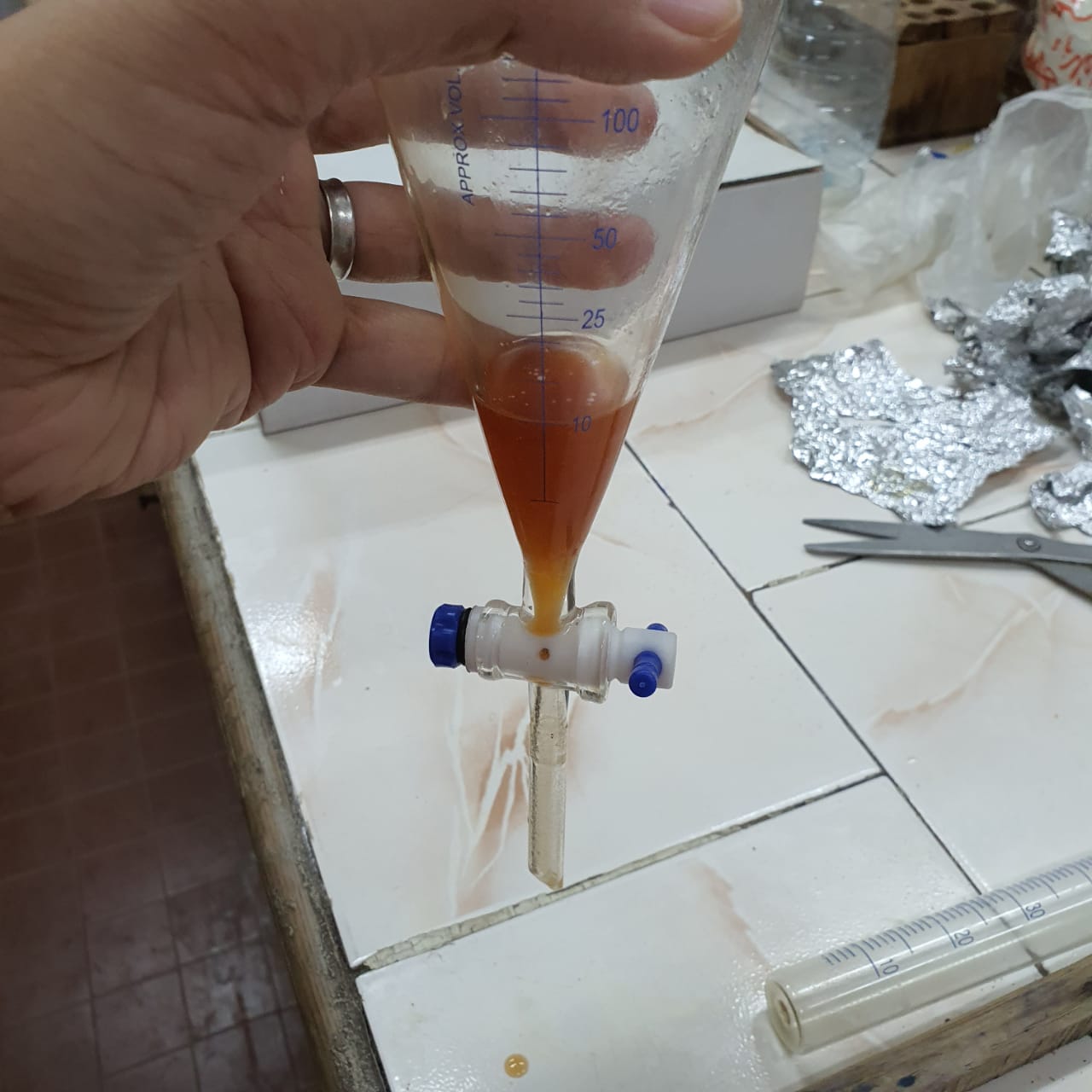


**Fig S2: Remaining FOW at the end of biosurfactant production experiment**
