# Supplementary material for: Valorization of frying oil waste for biodetergent production using Serratia marcescens N2 and gamma irradiation assisted biorecovery
Source: Microb Cell Fact. 2022 Jul 30;21:151. doi: 10.1186/s12934-022-01877-3 (PMC9338678; doi:10.1186/s12934-022-01877-3)
Supplement: Supplementary file 2 — Additional file 2: Table S1. Design of experiment. [file 12934_2022_1877_MOESM2_ESM.docx]

 Journal name: Journal of Biotechnology
- Manuscript Title: Valorization of oil waste for biodetergent production using *Serratia marcescens* N2 and gamma irradiation assisted biorecovery
Nora M. Elkenawy and Ola M. Gomaa^2^*

^1^Drug Radiation Research Department, ^2^Radiation Microbiology Department, National Center for Radiation Research and Technology (NCRRT), Egyptian Atomic Energy Authority (EAEA), Cairo-Egypt

*Corresponding author: ola_gomaa@hotmail.com

Table (S1): Design of experiment

| Std order | Run order | Pt type | blocks | Carbon source  % | Inoculum size  % |
| --- | --- | --- | --- | --- | --- |
| 4 | 1 | 1 | 1 | 10 | 2 |
| 2 | 2 | 1 | 1 | 5 | 4 |
| 5 | 3 | 1 | 1 | 10 | 4 |
| 7 | 4 | 1 | 1 | 20 | 2 |
| 6 | 5 | 1 | 1 | 10 | 8 |
| 1 | 6 | 1 | 1 | 5 | 2 |
| 9 | 7 | 1 | 1 | 20 | 8 |
| 8 | 8 | 1 | 1 | 20 | 4 |
| 3 | 9 | 1 | 2 | 5 | 8 |
